# Modified Prostate Health Index Density Significantly Improves Clinically Significant Prostate Cancer (csPCa) Detection

**DOI:** 10.3389/fonc.2022.864111

**Published:** 2022-04-07

**Authors:** Haojie Chen, Yuhang Qian, Yanyuan Wu, Bowen Shi, Jiatong Zhou, Fajun Qu, Zhengqin Gu, Jie Ding, Yongjiang Yu

**Affiliations:** Department of Urology, School of Medicine, Xinhua Hospital Affiliated to Shanghai Jiao Tong University, Shanghai, China

**Keywords:** clinically significant prostate cancer, PSA, gray zone, prostate health index density, mPHI

## Abstract

**Background:**

Early screening of clinically significant prostate cancer (csPCa) may offer opportunities in revolutionizing the survival benefits of this lethal disease. We sought to introduce a modified prostate health index density (mPHI) model using imaging indicators and to compare its diagnostic performance for early detection of occult onset csPCa within the prostate-specific antigen (PSA) gray zone with that of PHI and PHID.

**Methods and Participation:**

Between August 2020 and January 2022, a training cohort of 278 patients (total PSA 4.0–10.0 ng/ml) who were scheduled for a prostate biopsy were prospectively recruited. PHI and PHID were compared with mPHI $\left(\frac{\text{LD}}{\text{TRD}\times \text{APD}\times \text{TPV}}\times \text{PHI}\right)$ for the diagnosis performance in identifying csPCa. Pathology outcomes from systematic prostate biopsies were considered the gold standard.

**Results:**

This model was tested in a training cohort consisting of 73 csPCa, 14 non-clinically significant prostate cancer(non-csPCa), and 191 benign prostatic hyperplasia (BPH) samples. In the univariate analysis for the PSA gray zone cohort, for overall PCa, the AUC of mPHI (0.856) was higher than PHI (0.774) and PHID (0.835). For csPCa, the AUC of mPHI (0.859) also surpassed PHI (0.787) and PHID (0.825). For detection of csPCa, compared with lower specificities from PHI and PHID, mPHI performed the highest specificity (76.5%), by sparing 60.0% of unnecessary biopsies at the cost of missing 11 cases of csPCa. The mPHI outperformed PHI and PHID for overall PCa detection. In terms of csPCa, mPHI exceeds diagnostic performance with a better net benefit in decision curve analysis (DCA) compared with PHI or PHID.

**Conclusions:**

We have developed a modified PHI density (mPHI) model that can sensitively distinguish early-stage csPCa patients within the PSA gray zone.

**Clinical Trial Registration:**

ClinicalTrials.gov, NCT04251546.

## Introduction

Clinically significant prostate cancer (csPCa) has a high prevalence in older males and is even higher in those with a family history of the disease (1). Occult onset as a feature of csPCa usually ends with the progress of aggressive status and poor prognosis, ultimately causing huge consequences for the individuals and a hard socio-economic burden (2). The widespread screening of prostate-specific antigen (PSA) serum and system development of cancer activity surveillance make it possible to detect csPCa and intervene early to benefit the patients (3). However, early screening of prostate cancer (PCa) based on serum PSA has been the subject of great controversy due to its insufficient specificity and the risk of overdiagnosis and overtreatment, especially for “PSA gray zone” (total PSA ranging from 4 to 10 ng/ml) suspicious patients (4–6). Given the recent research about gray zone PCa incidence, it has been reported that only about 25% of men are finally diagnosed with PCa (7), and only about half are identified as having csPCa (8), meaning that more than 80% of patients have received unnecessary invasive biopsies. China, Japan, and South Korea, for example, belong to the high incidence rate regions for PCa in Asia (9–11), with a higher incidence of PCa within the PSA gray zone than other Asian countries; despite benefitting from early screening diagnosis followed by active intervention and maintaining a remarkable 5-year survival rate compared with other Asian countries, they are also suffering a substantial wasting of medical resources. The majority of initially suspected patients with abnormal tPSA levels were finally confirmed to have indolent non-clinical significant prostate cancer (non-csPCa) or even benign disease, which would have a practical impact on patients during their lifetimes (12, 13). Thus, to improve the specificity of detection for csPCa and avoid overdiagnosis of negative/non-csPCa within the PSA gray zone, new strategies are urgently required.

To achieve this purpose, novel predictors have emerged to reform the diagnosis strategy, namely, serum test-based biomarkers like free PSA (fPSA), [−2] pro prostate-specific antigen (p2PSA), %fPSA, %p2PSA, and the prostate health index (PHI) (14–16). Furthermore, with the growing availability of TRUS and mpMRI in the detection of prostate cancer, more studies have reported the utility of the combination of serum biomarkers and imaging assessment. Imaging-assessment-based predictor PSA density (PSAD) was once regarded as one of the promising strategies (17). The underlying mechanism of PSAD for csPCa detection was that malignant cells might generate extra PSA more than an equal volume of the normal gland (18). More recently, PHI density (PHID) was developed drawing on the concept of PSAD and combined with serum-based predictor PHI (19). While limited as a diagnostic tool, PHID has been validated by several clinical studies to demonstrate good diagnostic performance, and it has been shown to have superior predictive accuracy compared with any other PSA-derives as one of the most advanced csPCa predictors (20, 21).

However, due to the highly heterogeneous nature of csPCa and large individual differences among patients within the PSA gray zone (22), it is unreasonable to rely on the total prostate volume (tPV) as an image standardization alone. It is necessary to comprehensively consider the situation of the patient and then fit different image parameters to optimize the predicting model. Currently, though PHID has been proven to better identify PCa within the PSA gray zone (23), the modality still possesses limitations. Above all, bias in results will come upon when using the tPV as a normalized control for PHID or PSAD. The prostate is a three-dimensional organ with mainly three volume diameters (transverse diameter, TRD; longitudinal diameter, LD and anteroposterior diameter, APD) measured by TRUS or mpMRI (24). Patients with different tumor heterogeneity may have the same calculated results of tPV values and PHI levels despite containing different three-dimensional structures with distinct diameters. Therefore, the same PHID score is obtained, which cannot be used to differentiate tumor heterogeneity between csPCa and non-csPCa, eventually leading to misdiagnosis of malignant or overdiagnosis of benign disease.

Enlightened by the thyroid cancer screening (25), our group recently investigated the ability of the three diameters mentioned above to adjust the PHID predictor and expected it to show better distinguishing ability and predictive value for early-stage csPCa. According to our unpublished data, prostate LD might increase faster than TRD and APD in csPCa, which may be due to the active proliferation of malignant cells in the peripheral zone (26). This is because it is imperative to explore better strategies for reducing csPCa overdiagnosis and avoiding unnecessary biopsies within the gray zone. Similarly to how PSAD and PHID were derived, and based on the familiar logical mechanism, we hypothesized LD as a tumor occurrence positive factor (numerator) and TRD, APD, and prostate volume as negative factors (denominator) for PHI predictor modification and provided a preliminary novel predictor mPHI, $\left(\frac{\text{LD}}{\text{TRD}\times \text{APD}\times \text{TPV}}\times \text{PHI}\right)$ . The main objective of the present study is to evaluate the clinical utility of mPHI in csPCa identifying compared with PHI and PHID.

## Materials and Methods

### Study Design and Population

This study was a prospective, observational single-center study. A prospective cohort consisting of 310 men who were scheduled to undergo an initial biopsy for suspicious PCa due to an elevated tPSA ranging between 4.0–10.0 ng/ml without abnormal findings on DRE was recruited in our institution between August 2020 and January 2022. PHI detection and transrectal ultrasonography (TRUS) were performed, followed by systematic prostate biopsies. Patients were excluded in the present study if they (1) had previous histories of prostate cancer, prostate biopsy, 5-α reductase inhibitors treatment, or an inability to sign informed consent or (2) the records of any serum antigen levels (tPSA, fPSA, or p2PSA) and image parameter (tPV, LD, TRD, and APD) were missing.

A total of 278 patients with written informed consent were finally enrolled: 73 had clinically significant prostate cancer (csPCa) and 14 had non-clinically significant prostate cancer (non-csPCa) confirmed by biopsy, while the rest of the 191 patients were benign.

### Methods

After obtaining written informed consent, blood samples were collected before prostate biopsy and stored at −80°C after centrifugation. The serum samples were anonymized before storage. The tPSA, fPSA, and p2PSA levels were measured with the Beckman Coulter DxI800 Unicel Immunoassay system. PHI was determined according to the formula: PHI = (p2PSAfree PSA×(tPSA)12) . Total prostate volume was calculated with TRUS or MRI using the standard ellipsoid formula along with transverse diameter (TRD), longitudinal diameter (LD), and anteroposterior diameter (APD) measurement. To verify the hypothesis that LD positively correlated to csPCa, we proposed a modified PHI (mPHI) formula that combined the image parameters and PHI: mPHI $\left(\frac{\text{LD}}{\text{TRD}\times \text{APD}\times \text{TPV}}\times \text{PHI}\right)$ .

Prostate biopsy specimens were examined and assessed by experienced and skilled pathologists, while the Gleason scores were graded according to the 2014 International Society of Urological Pathology Consensus Conference (27). All patients took a biopsy guide by TRUS with at least 12 cores with or without radical prostatectomy. Moreover, clinically significant PCa (csPCa) was defined as a Gleason score ≥7, or a Gleason score of 6 but with ≥3 positive cores and/or a maximum core involvement of ≥50% according to Epstein’s criteria (28).

The primary endpoint was to evaluate the diagnostic accuracy and ability to avoid unnecessary biopsy of mPHI in identifying the presence of csPCa within the PSA gray zone in comparison to PHI and PHID.

### Statistical Analysis

Pathology outcomes from systematic prostate biopsies were considered the gold standard. Univariable logistic regression analysis was used to determine the association between covariates and csPCa performed by the “generalized linear model” function with binomial parameter in R. The receiver operating characteristic (ROC) curve analysis and area under the receiver operating characteristic curve (AUC) was used to examine the sensitivity, specificity, and diagnostic accuracy of mPHI in comparison with PHI and PHID for early detection of csPCa in the PSA gray zone.

The predicted avoided prostate biopsy number was compared. Decision curve analysis (DCA) was performed by the “ggDCA” (version 1.1) and “rms” (version 6.2) R packages and used to compare the predictive accuracy of mPHI with PHI and PHID. The analysis was conducted by R (version 4.0.0) and MedCalc (version 15.2.2). Comparison between ROC curves was performed by MedCalc.

## Results

**Figure 1:**
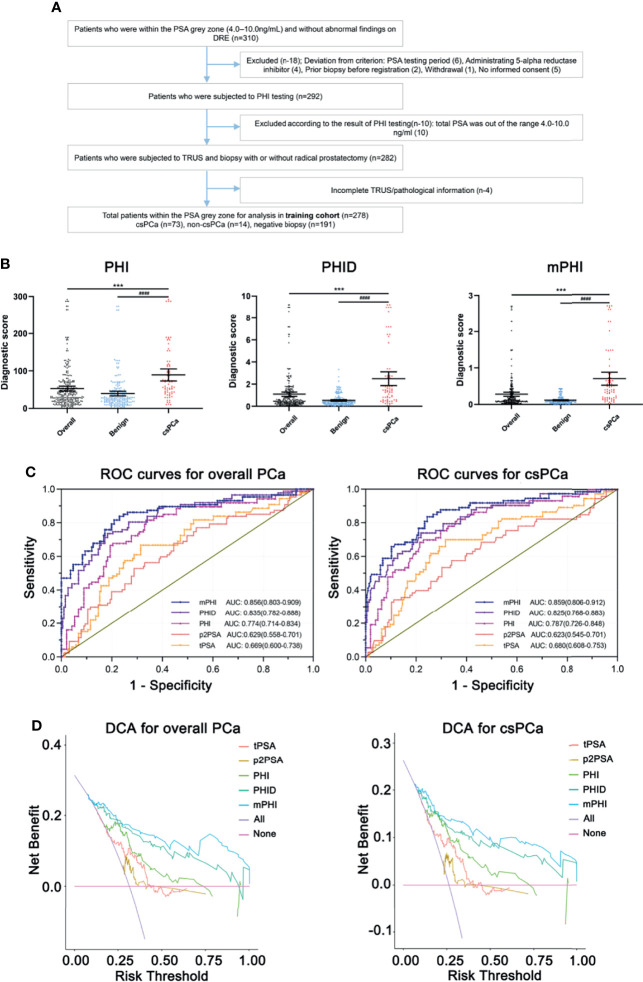
**(A)** Flow diagram of enrolled participants of the training cohort. **(B)** Diagnostic scores of PHI, PHID, and mPHI in clinically significant prostate cancer and benign biopsies identification. *Overall vs csPCa, ^#^Benign vs csPCa. Data are shown as Mean ± SEM. ***P <0.001, ^####^P <0.0001. **(C)** Receiver operating analysis (ROC) curve of the training cohort show the area under the ROC curve for overall PCa (left) and clinically significant prostate cancer (right) for the prediction of clinically significant prostate cancer on prostate biopsy, namely, tPSA, total PSA, p2PSA, [−2] pro-PSA, PHI, prostate health index, PHID, PHI density, mPHI, defined as $\left(\frac{\text{LD}}{\text{TRD}\times \text{APD}\times \text{TPV}}\times \text{PHI}\right)$ . **(D)** Decision-curve analysis showing the net benefit and diagnostic performance of each prediction model in the training cohort for overall PCa (left) and clinically significant prostate cancer (right). The modified Prostate Health Index (mPHI) score (top line) demonstrated separation from the other biomarkers across a wide range of threshold values tPSA, total PSA; p2PSA, [−2] pro-PSA; PHI, prostate health index; PHID, PHI density; mPHI, modified PHI.

Overall, 278 of the 310 registered men (89.6%) who were within the PSA gray zone and met the study criteria were finally recruited and formed the training cohort (**Figure 1A**): 73 csPCa (26.2%), 14 non-csPCa (5.0%), and 191 negative biopsies (68.7%). **Table 1** shows the population characteristics of the study. As for serum biomarkers, men with csPCa showed there was no significance in age compared to overall patients (P = 0.0528). Meanwhile, the median total prostate volume is higher in those with negative biopsies or non-clinically significant PCa (80.36 vs 36.31), and the levels of LD, APD, and TRD of overall patients are all higher than those of csPCa, which may be due to the major proportion of benign prostatic hyperplasia (BPH) with larger tPV in patients with negative biopsies. Particularly, compared with those who are pathologically confirmed with negative or non-csPCa, the csPCa cases show higher PHI, PHID, and mPHI (all P <0.0001). Of the presented predictors, significant differences exist in men with vs without clinically significant cancer.

**Table 1:** Clinical characteristics of the study cohorts within PSA gray zone.

Median (IQR)	Overall (n = 278)	Clinically significant	Negative or non-clinically significant PCa (n = 205)	*P*
		PCa (n = 73)		
**Age, years**	69 (65–73)	67 (63–71)	70 (65–75)	0.0528
**tPSA, ng/ml**	7.27 (5.74–9.19)	8.98 (6.95–9.85)	6.95 (5.53–8.47)	0.0009
**fPSA, ng/ml**	1.10 (0.81–1.27)	0.91 (0.61–1.26)	1.15 (0.90–1.91)	0.0042
**p2PSA, pg/ml**	15.02 (8.83–24.88)	20.79 (12.14–33.73)	13.86 (8.61–23.92)	0.0014
**LD, cm**	5.6 (4.8–6.2)	5.1 (3.96–5.66)	6.0 (5.3–6.3)	<0.0001
**TRD, cm**	5.4 (5.0–5.9)	4.9 (4.5–5.4)	5.5 (5.2–5.9)	<0.0001
**APD, cm**	4.4 (3.8–4.9)	3.5 (3.0–4.2)	4.6 (4.1–5.0)	<0.0001
**TPV, ml**	68.88 (47.92–90.16)	36.31 (29.95–56.01)	80.36 (62.00–97.37)	<0.0001
**PHI**	36.67 (19.83–65.22)	70.97 (43.70–115.66)	28.42 (17.21–47.48)	<0.0001
**PHID**	0.50 (0.27–1.28)	1.49 (0.59–2.74)	0.40 (0.23–0.65)	<0.0001
**mPHI**	0.11 (0.06–0.30)	0.40 (0.17–0.75)	0.08 (0.05–0.13)	<0.0001

TPV, total prostate volume; LD, longitudinal diameter; TRD, transverse diameter; APD, anteroposterior diameter; tPSA, total PSA; fPSA, free PSA; p2PSA, [-2] pro-PSA; PHI, prostate health index, PHI = [(p2PSA/free PSA) × (PSA)½]; PHID, PHI density; mPHI, defined as $\left(\frac{\text{LD}}{\text{TRD}\times \text{APD}\times \text{TPV}}\times \text{PHI}\right)$ ; *IQR, interquartile range; Data are given as median (IQR), unless otherwise indicated. P-value shows the significance between overall and clinically significant PCa in each cohort.*

In terms of univariable logistic regression (**Table 2**), image indexes and serum biomarker tPSA and fPSA, except for p2PSA (P = 0.087), are all associated with clinically significant PCa. Older age fails to demonstrate overall PCa (P = 0.053) but is a predictor for csPCa (P = 0.015). By contrast, volume-related parameters (namely, TPV, LD, TRD, APD) are all associated with both PCa and csPCa (P <0.001). Moreover, PHI, PHID, and mPHI appear to be the most important predictors of PCa and csPCa (all P <0.001). Notably, each one-point increase in PHID and mPHI is associated with a more than four-fold increase in the odds of csPCa on biopsy or a more than five-fold in the odds of overall PCa. Diagnostic scores of PHI, PHID, and mPHI in clinically significant prostate cancer and benign biopsies identification represent their prediction ability (**Figure 1B**).

**Table 2:** Univariable logistic regression models for the PSA gray zone clinically significant PCa prediction.

	Overall (n = 278)		Clinically significant PCa (n = 73)	
	OR (95% CI) per unit increase	*P*	OR (95% CI) per unit increase	*P*
**age, years**	0.96 (0.93–1)	0.053	0.95 (0.91–0.99)	0.015
**tPSA, ng/ml**	1.26 (1.12–1.4)	<0.001	1.28 (1.14–1.44)	<0.001
**fPSA, ng/ml**	0.44 (0.29–0.67)	<0.001	0.45 (0.29–0.71)	<0.001
**p2PSA, pg/ml**	1.01 (1–1.02)	0.103	1.01 (1–1.02)	0.087
**LD, cm**	0.12 (0.07–0.19)	<0.001	0.15 (0.09–0.24)	<0.001
**TRD, cm**	0.22 (0.14–0.36)	<0.001	0.21 (0.13–0.36)	<0.001
**APD, cm**	0.12 (0.07–0.21)	<0.001	0.16 (0.1–0.25)	<0.001
**TPV, ml**	0.94 (0.92–0.95)	<0.001	0.94 (0.93–0.96)	<0.001
**PHI**	1.02 (1.01–1.02)	<0.001	1.02 (1.01–1.02)	<0.001
**PHID**	5.32 (3.27–8.65)	<0.001	4.33 (2.75–6.81)	<0.001
**mPHI**	3,548.98 (410.65–30,671.88)	<0.001	976.79 (143.35–6,655.68)	<0.001

OR, odds ratio; CI, confidence interval; TPV, total prostate volume; LD, longitudinal diameter; TRD, transverse diameter; APD, anteroposterior diameter; tPSA, total PSA; fPSA, free PSA; p2PSA, [−2] pro-PSA; %fPSA, free PSA/total PSA; %p2PSA, p2PSA/free PSA; PSAD, PSA density; PHI, prostate health index, PHI = [(p2PSA/free PSA) × (PSA)½]; PHID, PHI density; mPHI, defined as $\left(\frac{\text{LD}}{\text{TRD}\times \text{APD}\times \text{TPV}}\times \text{PHI}\right)$ .

The area under curves (AUC) is applied to measure the utility of every diagnostic tool (tPSA, p2PSA, PHI, PHID, and mPHI) to discriminate overall PCa and csPCa (**Figure 1C**). Briefly, the single-serum biomarker tPSA and p2PSA models yield AUCs of 0.669 and 0.629 in overall PCa prediction while 0.680 and 0.623 for csPCa prediction, which shows a relatively unsatisfactory predictive capability. Meanwhile, the integrated models (PHI, PHID, and mPHI) yield AUCs for detection of PCa and csPCa are as follows: PHI (AUC = 0.774 and 0.787), PHID (AUC = 0.835 and 0.825), mPHI (AUC = 0.856 and 0.859). Among the tested biomarkers, the AUC of mPHI still outperforms PHI or PHID. Notably, decision-curve analysis (DCA) suggests a superior net benefit of the mPHI model over various diagnostic strategies (**Figure 1D**). Furthermore, csPCa diagnosis models are listed below in order of net benefit from most to least: mPHI, PHID, PHI, p2PSA, and tPSA (threshold ranging from 30 to 50%). The same order appears for the diagnosis performance of PCa. As is consistent with the AUC results, mPHI has the most improvement in a clinical net benefit for both PCa and csPCa within the PSA gray zone. Moreover, the expected ratio of biopsy being avoided and the ratio of csPCa being missed of each predictor at the best cut-offs are listed in **Table 3**. The diagnostic performance of mPHI surpasses PHI and PHID by avoiding unnecessary biopsies of 167 patients at the cost of 11 PCa patients being missed while maintaining 80.8% sensitivity and 76.5% specificity in the study cohort. The mPHI outperforms any other predictors, avoids maximum unnecessary biopsies at the least cost of csPC missed% while maintaining the highest diagnostic sensitivity and specificity for csPCa. Similar results appear in the overall PCa diagnosis, indicating that mPHI is universally applicable with an outperformer diagnostic ability than PHI and PHID.

**Table 3:** Diagnostic performance of prostate cancer predictors at the best cut-off values.

Overall prostate cancer (PCa)						
Predictor	Cut-off	Sensitivity/Specificity, %	PPV/NPV, %	Biopsy avoided %	PCa missed %	*P*
**tPSA**	>7.691	66.67/68.59	49.2/81.9	56.8 (158/278)	33.3 (29/87)	<0.0001
**p2PSA**	>20.551	52.87/71.20	45.5/76.8	62.9 (175/278)	47.1 (41/87)	0.0004
**PHI**	>36.952	81.61/66.49	52.6/88.8	50.3 (140/278)	18.3 (16/87)	<0.0001
**PHID**	>0.776	75.86/81.68	65.3/88.1	56.1 (156/278)	24.1 (21/87)	<0.0001
**mPHI**	>0.128	83.91/82.96	62.4/91.3	56.8 (158/278)	16.0 (14/87)	<0.0001
**Clinically significant prostate cancer (csPCa)**						
**Predictor**	**Cut-off**	**Sensitivity/Specificity, %**	**PPV/NPV, %**	**Biopsy avoided %**	**csPCa missed %**	***P***
**tPSA**	>7.743	69.86/68.29	44.0/86.4	57.5 (160/278)	30.1 (22/73)	<0.0001
**p2PSA**	>18.482	57.53/65.85	37.5/81.3	59.3 (165/278)	42.4 (31/73)	0.0021
**PHI**	>34.631	83.56/60.49	43.0/91.2	48.2 (134/278)	16.4 (12/73)	<0.0001
**PHID**	>0.713	76.71/75.61	52.8/90.1	57.5 (160/278)	23.2 (17/73)	<0.0001
**mPHI**	>0.139	80.82/76.59	55.1/91.8	60.0 (167/278)	15.0 (11/73)	<0.0001

PPV, positive predictive value; NPV, negative predictive value; PHI, prostate health index, PHI = [(p2PSA/free PSA) × (PSA)½]; PHID, PHI density; mPHI, defined as $\left(\frac{\text{LD}}{\text{TRD}\times \text{APD}\times \text{TPV}}\times \text{PHI}\right)$ ; *Biopsy avoided was restricted to patients with predictor value ≤cut-off and divided by the number of total enrolled patients while PCa missed refers to patients with the same conditions but divided by the number of pathological confirmations in each cohort. Data are given as a percentage (%), unless otherwise indicated.*

With regard of the diagnostic performance of mPHI, an NPV of 91.8% for the detection of csPCa and an NPV of 91.3% for overall PCa detection is performed. According to the best cut-off value of mPHI, prostate biopsies can be avoided by 56.8% (158/278) of overall participants at the cost of 16.0% (14/87) PCa patients being missed, while 60.0% (167/278) can be avoided at the cost of 15.0% (11/73) csPCa being missed. Therefore, we preliminary illustrates that the image parameter modified PHI predictor (mPHI) can avoid about 55–60% of unnecessary biopsies at the cost of 15% missed diagnosis while maintaining about 80% sensitivity and 75% specificity in csPCa detection. Despite this, the best cut-off value needs to be adjusted in a large-sample, multicenter study.

## Discussion

The primary objective of our present research was to assess the combination between volume diameters and PHI and to determine whether the modified PHI (mPHI) would provide additional predictive value than PHI and PHID in identifying gray zone csPCa. Socioeconomic acknowledgment of PCa overdiagnosis has given impetus to alter the priorities, from overall cancer diagnosis to prioritizing accurate detection of the PSA gray zone cancers. Among them, the critical driving factor of this impetus is the balance of risks and cost-effectiveness for widespread gray zone csPCa screening. Furthermore, concerning the COVID-19 pandemic, it is important to discuss the influence on the clinical management of prostate cancer. The opinions of Crocetto and Di Lorenzo describe the clinical characteristics of prostate cancer patients who are COVID-19 positive (29, 30). Although serum PSA screening for early PCa detection has been widespread in China, our study started in 2020, when the COVID-19 pandemic had begun. China adopted a strict public health control policy that might affect how people with abnormal PSA seek medical advice and further treatment. Some patients with slightly elevated PSA might stay at home; others whose PSA value has been steadily rising for a certain period are probably more willing to take biopsies.

Studies on using PHI as a gray zone prostate cancer detection tool have been emerging over the past decade, considerably benefitting the avoidance of overdiagnosis and overtreatment (31, 32). Moreover, several studies have discussed the emerging landscape of PHI as a novel tumor marker (33, 34). Considering the combination of serum markers and imaging markers, a recent study (35) demonstrates that prostate volume (PV) does not show additional value for PHI-PV derivatives (PHID or PHIV) in PCa or csPCa prediction. Huang et al. (35) believe that the reason is that p2PSA, as an essential component of PHI, is mainly expressed in tumor tissue, which means tumor volume is more reasonable than PV as the denominator part of PHID. Besides, both tPSA and fPSA are positively linearly associated with tPV. The formation of the PHID (p2PSA×(tPSA)12PV×fPSA) itself already contains (tPSA)12fPSA , which may result in a naturally weakened contribution provided by tPV. Besides, they attempt to improve the structure of PHID and introduce the concept of PHIV ( PHI(PV)12 ), but fail to get a satisfying result. Altogether, after comprehensive analysis between risks and benefits, PHID is not recommended as a diagnostic tool for PCa and widespread csPCa screening due to its little additional predictive value.

With regards to this, we planned and arranged to reassemble and optimize existing diagnostic prediction models, namely, p2PSA, PHI, and PHID, and attempted to balance the benefit and risk. Recent research has proved that the modified PHI predictive model outperforms PHI density in the early distinguishing of overall prostate cancer and BPH (36). Based on the logical mechanism of Huang et al., we supposed that LD might increase faster than TRD and APD in csPCa due to the active proliferation of malignant cells in the peripheral zone, which mainly composed the LD measurement. Since the tumor area in which p2PSA is highly expressed is virtually indistinguishable by imaging examination at an early stage, “tumor volume” is an impossible parameter. Instead, the LD of total prostate cancer can be approximated regarded as a replacement of the “tumor volume”, and as an existence index, it is usually measured by most TRUS and MRI. No additional examination increasing the burden for early screening is required. Therefore, we sought to incorporate all parameters, namely, tPV, LD, TRD, and APD which already exist in TRUS or mpMRI, and optimized their combination form to maximize diagnostic performance by a new predicting model as modified PHI.

Obviously, the diagnosis of clinical prostate cancer is no longer the acceptable standard but the finding of clinically significant prostate cancer (csPCa) is the new clinical benchmark for early detection. Our mPHI risk predictor has a higher AUC than previous studies and can greatly reduce the number of unnecessary biopsies, establishing an improved combination of prostate volume and diameter indexes and PHI, compared to PHID (considered to be the best predictor published as yet), contributing optimized risk–benefit and detection utility for cancer screening. No additional test is necessary. The presented modified PHI risk predictor may be introduced into not only developed countries but also developing nations (37).

Since this is the first prospective research with finite case numbers from a single center to assess the influence of the volume diameters on the adjustment of PHI predictor, several limitations and biases might exist in this study. Despite the preliminary test we have given the improved model with the highest AUC presented so far, generalizability was restricted by the single ethnicity of participants, local environmental factors, and so on. There is no doubt that a deeper optimization version is urgently needed. In addition, another major limitation of this study was that image parameters, namely, tPV, LD, TRD, and APD were all measured by the same type of transrectal approach (TRUS) or MRI, and despite all data being measured by experienced ultrasonologists, biases might exist. Moreover, the result of the diagnostic performance of mPHI model may be limited by patient number and require further validation and improvement to assess the capability of mPHI risk predictor in gray zone csPCa identification in large-sample, multicenter studies.

### Conclusions

In our single-center cohort, we found that the modified PHI (mPHI) $\left(\frac{\text{LD}}{\text{TRD}\times \text{APD}\times \text{TPV}}\times \text{PHI}\right)$ predictor outperformed PHI and PHID and could reduce more unnecessary biopsies in csPCa early detection within the PSA gray zone.

## Data Availability Statement

The raw data supporting the conclusions of this article will be made available by the authors, without undue reservation.

## Ethics Statement

The studies involving human participants were reviewed and approved by the Ethics Committee of Xinhua Hospital affiliated to Shanghai Jiao Tong University School of Medicine (XHEC-C-2019-113-2). The patients/participants provided their written informed consent to participate in this study.

## Author Contributions

Conception and design: YY and JD. Acquisition of data: YQ. Manuscript writing: HC and BS. Data management and analysis: YW and JZ. Manuscript editing: ZG and FQ. All authors listed have made a substantial, direct, and intellectual contribution to the work and approved it for publication.

## Funding

This work was supported by the Clinical Research Plan of SHDC (grant no. SHDC2020CR4034) and the Xinhua Hospital Clinical Innovation Fund (grant no. 19XHCR01A).

## Conflict of Interest

The authors declare that the research was conducted in the absence of any commercial or financial relationships that could be construed as a potential conflict of interest.

## Publisher’s Note

All claims expressed in this article are solely those of the authors and do not necessarily represent those of their affiliated organizations, or those of the publisher, the editors and the reviewers. Any product that may be evaluated in this article, or claim that may be made by its manufacturer, is not guaranteed or endorsed by the publisher.

## Abbreviations

mPHI, modified prostate health index density; PHI, prostate health index;PHID, prostate health index density; PSA, prostate specific antigen; PCa, prostate cancer; csPCa, clinically significant prostate cancer; non-csPCa, non-clinically significant prostate cancer BPH, benign prostatic hyperplasia; tPSA, total prostate specific antigen; fPSA, free PSA; p2PSA, [−2] pro PSA; TPV, total prostate volume; LD, longitudinal diameter; TRD, transverse diameter; APD, anteroposterior diameter; AUC, area under curve; ROC, receiver operating characteristic curve; DCA, decision curve analysis; DRE, digital rectal examination; mpMRI, multiparametric magnetic resonance imaging; TRUS, transrectal ultrasonography.
